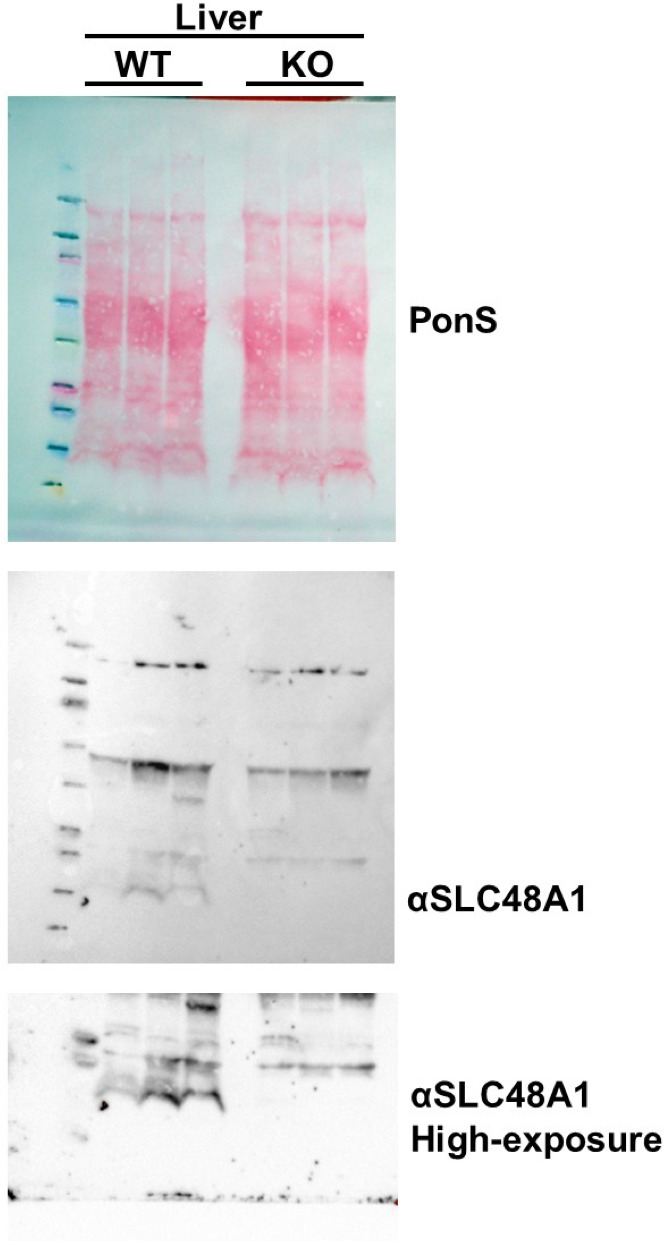# Correction: Hemozoin produced by mammals confers heme tolerance

**DOI:** 10.7554/eLife.93253

**Published:** 2023-10-20

**Authors:** Rini H Pek, Xiaojing Yuan, Nicole Rietzschel, Jianbing Zhang, Laurie Jackson, Eiji Nishibori, Ana Ribeiro, William Simmons, Jaya Jagadeesh, Hiroshi Sugimoto, Md Zahidul Alam, Lisa Garrett, Malay Haldar, Martina Ralle, John D Phillips, David M Bodine, Iqbal Hamza

**Keywords:** Mouse

Pek RH, Yuan X, Rietzsche N, Zhang J, Jackson L, Nishibori E, Ribeiro A, Simmons W, Jagadeesh J, Sugimoto H, Alam MZ, Garrett L, Haldar M, Ralle M, Phillips JD, Bodine DM, Hamza I. 2019. Hemozoin produced by mammals confers heme tolerance. *eLife* **8**:e49503. doi: 10.7554/eLife.49503.

Published 1 October 2019

It was brought to our attention through PubPeer (PubPeer - Hemozoin produced by mammals confers heme tolerance) that we duplicated the loading controls of the spleen Western Blots for the liver in Fig. 1C. After checking the first author’s lab notebooks and the raw figure files, we found the original images for the liver samples, which show the correct ponceau S stained nitrocellulose membranes. We can confirm that the duplication was inadvertent, and we have now corrected Fig. 1C by using the correct ponceau S staining image. During carefully proofreading the manuscript, we identified two textual errors in Figure legends 4 and 6. These changes neither impact the results nor the conclusions in the manuscript.

1. The correct figure legend for Figure 4—figure supplement 1 should be “…Gating of (**C**) bone marrow Ter-119^+^populations II+III and (**D**) splenic Ter-119^+^subpopulations.”

The original figure legend stated “…Gating of (**C**) splenic Ter-119^+^ populations II+III and (**D**) bone marrow Ter-119^+^ subpopulations.”

2. The correct figure legend for Figure 6—figure supplement 1 should be “…(**A**) Representative images of intracellular reactive oxygen species (ROS) in WT and KO BMDMs at the indicated timepoints post-treatments. (**B**) Representative images of WT and KO BMDMs post-EP.”

The original figure legend was “…(**A**) Representative images of WT and KO BMDMs post-EP. (**B**) Representative images of intracellular reactive oxygen species (ROS) in WT and KO BMDMs at the indicated timepoints post-treatments.”

The corrected figure 1C is shown below:

**Figure 1:**
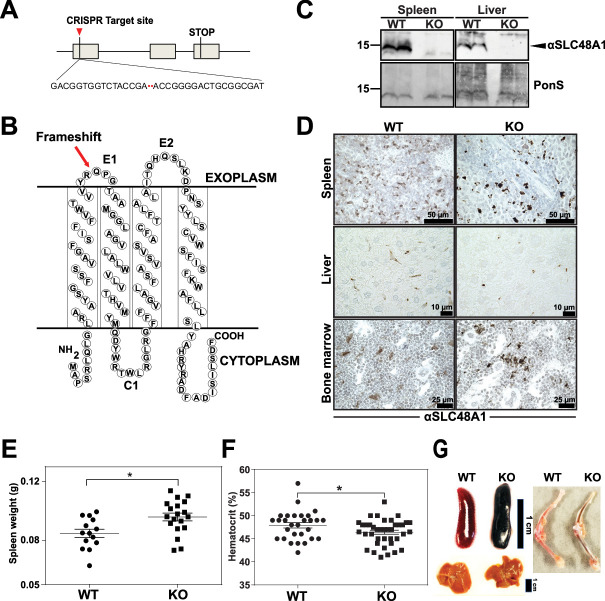


The originally published Figure 1 is shown for reference:

**Figure 2:**
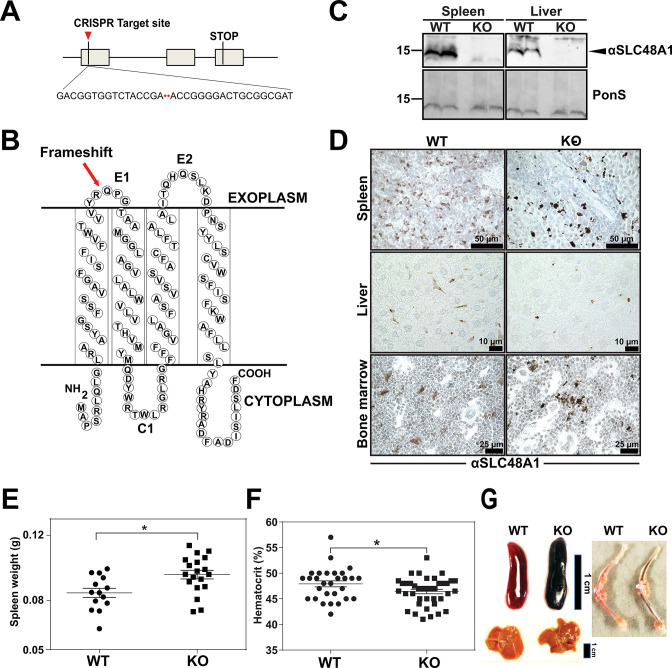


Here are the source images for reference.

**Figure 3:**