# Supplementary material for: Hemozoin produced by mammals confers heme tolerance
Source: eLife. 2019 Oct 1;8:e49503. doi: 10.7554/eLife.49503 (PMC6773446; doi:10.7554/eLife.49503)
Supplement: Supplementary file 1. — Table 2: Serum iron panel for WT and KO animals fed standard or 2ppm iron diet. Table 3: qRT-PCR analyses for iron/heme metabolism genes. [file elife-49503-supp1.docx]

**Supplementary Table 1: SLC48A1/HRG1 Mutant alleles produced by CRISPR/Cas9**

| **Mutant Name** | **Strain** | **Mutation** | **Sequence** |
| --- | --- | --- | --- |
| M1 | SVJ129/C57BL6J | 7bp deletion | …GACGGTGGTCTACCG•••••••GGGACTGCGGCGAT… |
| M2 | SVJ129/C57BL6J | 7bp deletion | …GACGGTGGTCTACCGA•••••••GGACTGCGGCGAT… |
| M3 | SVJ129/C57BL6J | 18bp deletion | …GACGG••••••••••••••••••GGACTGCGGCGAT… |
| M4 | SVJ129/C57BL6J | 1bp insertion | …GACGGTGGTCTACCGACAAACCGGGGACTGCGGCGAT… |
| M5 | SVJ129/C57BL6J | 1bp deletion | …GACGGTGGTCTACCGACA•CCGGGGACTGCGGCGAT… |
| M6 | SVJ129/C57BL6J | 2bp deletion | …GACGGTGGTCTACCGA••ACCGGGGACTGCGGCGAT… |
| M7 | SVJ129/C57BL6J | 11bp deletion | …GACGGTGG•••••••••••CCGGGGACTGCGGCGAT… |
| WT |  |  | …GACGGTGGTCTACCGACAACCGGGGACTGCGGCGAT… |

**Supplementary Table 2: Serum iron panel for WT and KO animals fed standard or 2ppm iron diet**

|  | **Standard diet** | | **2 ppm diet** | | ***N*** |
| --- | --- | --- | --- | --- | --- |
|  | **WT** | **KO** | **WT** | **KO** |  |
| **Serum iron (µg/dl)** | 113.5±11.72^a^ | 129.3±11.34^a^ | 66.32±13.62^b^ | 65.63±17.09^b^ | 8-15 |
| **TIBC (µg/dl)** | 348.4±32.1^a^ | 341.8±11.62^a^ | 451.8±17.08^b^ | 459±19.92^b^ | 8-15 |
| **Tf saturation (%)** | 36.05±4.452^a^ | 37.35±2.601^a^ | 14.3±2.975^b^ | 14.6±4.034^b^ | 8-15 |
| **Serum ferritin (ng/ml)** | 369.1±57.02^a^ | 585.9±58.62^b^ | 284.7±63.8^a^ | 492±63.77^b^ | 9-15 |

**Supplementary Table 3: qRT-PCR analyses for iron/heme metabolism genes.**

| **Spleen** | | | | | | | |
| --- | --- | --- | --- | --- | --- | --- | --- |
| **Standard** | | | | **2 ppm** | | | |
| Upregulated | p-value | Downregulated | p-value | Upregulated | p-value | Downregulated | p-value |
| Pcbp4 | 0.020129 | Slc48a1 | 0.023006 | Heph | 0.00032 |  |  |
|  |  | Cp | 0.040584 | Bmp6 | 0.00041 |  |  |
|  |  |  |  | Hfe2 | 0.000626 |  |  |
|  |  |  |  | Neo1 | 0.001396 |  |  |
|  |  |  |  | Il6 | 0.003406 |  |  |
|  |  |  |  | Pcbp4 | 0.003501 |  |  |
|  |  |  |  | Il10 | 0.004212 |  |  |
|  |  |  |  | Stat6 | 0.006443 |  |  |
|  |  |  |  | Hpx | 0.007156 |  |  |
|  |  |  |  | Steap1 | 0.00791 |  |  |
|  |  |  |  | Tmprss6 | 0.007936 |  |  |
|  |  |  |  | Ifna4 | 0.008373 |  |  |
|  |  |  |  | Saa1 | 0.008779 |  |  |
|  |  |  |  | Nox4 | 0.008779 |  |  |
|  |  |  |  | Epo | 0.008779 |  |  |
|  |  |  |  | Gast | 0.008779 |  |  |
|  |  |  |  | Il22 | 0.008779 |  |  |
|  |  |  |  | Hamp | 0.008779 |  |  |
|  |  |  |  | Nox3 | 0.008779 |  |  |
|  |  |  |  | Steap2 | 0.00879 |  |  |
|  |  |  |  | Nox1 | 0.008795 |  |  |
|  |  |  |  | Ifnb1 | 0.010902 |  |  |
|  |  |  |  | Asb11 | 0.011532 |  |  |
|  |  |  |  | Fbxl5 | 0.020012 |  |  |
|  |  |  |  | Tlr3 | 0.027149 |  |  |
|  |  |  |  | Pcbp3 | 0.027975 |  |  |
|  |  |  |  | Hfe | 0.037129 |  |  |
|  |  |  |  | Ifna2 | 0.041967 |  |  |
|  |  |  |  | Hp | 0.047671 |  |  |
|  |  |  |  | Cp | 0.050688 |  |  |
|  |  |  |  | Slc25a38 | 0.051437 |  |  |
|  |  |  |  | Slc38a1 | 0.054551 |  |  |
|  |  |  |  | Tlr7 | 0.05663 |  |  |
|  |  |  |  | Mfsd7b | 0.056827 |  |  |
|  |  |  |  | Hmox1 | 0.057685 |  |  |
| **Liver** | | | | | | | |
| **Standard** | | | | **2 ppm** |  |  |  |
| Upregulated | p-value | Downregulated | p-value | Upregulated | p-value | Downregulated | p-value |
| Ifna2 | 0.010002 | Slc48a1 | 0.004204 |  |  | Iscu | 0.004343 |
| Cybrd1 | 0.010447 |  |  |  |  | Slc48a1 | 0.004492 |
|  |  |  |  |  |  | Ncoa4 | 0.010102 |
|  |  |  |  |  |  | Slc11a2 | 0.01464 |
|  |  |  |  |  |  | Pcbp2 | 0.040291 |
