# Supplementary material for: Hemozoin produced by mammals confers heme tolerance
Source: eLife. 2019 Oct 1;8:e49503. doi: 10.7554/eLife.49503 (PMC6773446; doi:10.7554/eLife.49503)
Supplement: Transparent reporting form [file elife-49503-transrepform.pdf]

1st Floor
24 Hills Road
Cambridge CB2 1JP, UK

P 01223 855340
W elifesciences.org
T @elife

eLife's transparent reporting form

We encourage authors to provide detailed information *within their submission* to facilitate the interpretation and replication of experiments. Authors can upload supporting documentation to indicate the use of appropriate reporting guidelines for health-related research (see EQUATOR Network), life science research (see the BioSharing Information Resource), or the ARRIVE guidelines for reporting work involving animal research. Where applicable, authors should refer to any relevant reporting standards documents in this form.

If you have any questions, please consult our Journal Policies and/or contact us: editorial@elifesciences.org.

Sample-size estimation

- You should state whether an appropriate sample size was computed when the study was being designed
- You should state the statistical method of sample size computation and any required assumptions
- If no explicit power analysis was used, you should describe how you decided what sample (replicate) size (number) to use

Please outline where this information can be found within the submission (e.g., sections or figure legends), or explain why this information doesn't apply to your submission:

All mice studies were based on a power calculation as described below:
Power Calculation: The number of animals needed is estimated using a two-way ANOVA program with the following cutoff parameters: a) power of 0.80 b) alpha of 0.05 c) intragroup standard deviation of 15% d) detection threshold of 15% difference between groups. As applied to all of the proposed experiments, these parameters are fulfilled with 9 animals per experimental group. [Alpha: Level of significance set at 5%]: Ref:
http://www.ncbi.nlm.nih.gov/pmc/articles/PMC3826013/
https://www.ncbi.nlm.nih.gov/pubmed/?term=PMC3826013

Replicates

- You should report how often each experiment was performed
- You should include a definition of biological versus technical replication
- The data obtained should be provided and sufficient information should be provided to indicate the number of independent biological and/or technical replicates
- If you encountered any outliers, you should describe how these were handled
- Criteria for exclusion/inclusion of data should be clearly stated
- High-throughput sequence data should be uploaded before submission, with a private link for reviewers provided (these are available from both GEO and ArrayExpress)

Please outline where this information can be found within the submission (e.g., sections or figure legends), or explain why this information doesn't apply to your submission:

eLife Sciences Publications, Ltd is a limited liability non-profit non-stock corporation incorporated in the State of Delaware, USA, with company number 5030732, and is registered in the UK with company number FC030576 and branch number BR015634 at the address 1st Floor, 24 Hills Road, Cambridge CB2 1JP | August 2014

Biological replications are defined as the number of mice (n) used, or in *in vitro* experiments, biological replications are primary cells derived from different mice and experiments completed on a different day (i.e. how many times the experiment was done).
Technical replications are defined as the number of wells per cell culture dish for *in vitro* experiments, or the number of measurements made for each sample.
For XFM: biological replicates were considered as individual cells.

Outliers were identified using GraphPad PRISM's ROUT method (Robust regression and Outlier removal) (https://www.graphpad.com/guides/prism/7/curve-fitting/reg_rout_method_outliers.htm?toc=0&printWindow). Outliers were then excluded from subsequent analyses of the data.

Statistical reporting

- Statistical analysis methods should be described and justified
- Raw data should be presented in figures whenever informative to do so (typically when N per group is less than 10)
- For each experiment, you should identify the statistical tests used, exact values of N, definitions of center, methods of multiple test correction, and dispersion and precision measures (e.g., mean, median, SD, SEM, confidence intervals; and, for the major substantive results, a measure of effect size (e.g., Pearson's r, Cohen's d)
- Report exact p-values wherever possible alongside the summary statistics and 95% confidence intervals. These should be reported for all key questions and not only when the p-value is less than 0.05.

Please outline where this information can be found within the submission (e.g., sections or figure legends), or explain why this information doesn't apply to your submission:

All data are shown as means ± SEM unless otherwise stated. Means of groups were compared by using Student's unpaired t test. A p value of <0.05 was considered statistically significant. Analyses were performed using PRISM 7 software (GraphPad).

For all experiments involving mice, values of N are stated in the figure legends alongside the experiment it describes.

Figure 1
(E) spleen size: p=0.0036; 95% CI -0.01917 to -0.003254
(F) hematocrit: p=0.0374; 95% CI 0.08968 to 2.926

Supplemental Figure 1
(B) Chi-sq=2.404, p=0.3007

Figure 2
(B) BM RBC: p=0.0199; 95% CI 0.2584 to 9.687
(C) BM RBC populations: V (p=0.4790; 95% CI -5.76 to 6.06), IV (p=0.2402; 95% CI -4.476 to 2.195), III (p=0.4504; 95% CI -2.708 to 3.053), II (p=0.0243; 95% CI -3.947 to -0.01433), I (p=0.2673; 95% CI -3.479 to 1.872)
(E) Spleen RBC: p=0.1797; 95% CI -2.869 to 7.542)
(F) Spleen RBC populations: II+III (p=0.0205; 95% CI -8.864 to -0.2070)
(H) RPMs: p=0.0010; 95% CI 0.3550 to 1.379
(J) Monocytes ratio: p=0.0306; 95% CI -0.02830 to 1.130

Figure 3
In excel sheets (ICP; XFM)

Supplemental Figure 3
In excel sheets

Figure 4
(A) Kaplan-Meier survival: (Log-rank (Mantel-Cox test)) p<0.0001; Hazard Ratio (Mantel-Haenszel): 0.03906; 95% CI of ratio 0.007633 to 0.1999
(B) Hematocrit: Standard vs 2ppm: p value<0.0001; 95% CI 11.32 to 23.13 (WT); 15.95 to 25.80 (KO). WT vs KO: Standard p=0.2250; 95% CI -2.126 to 4.625. WT vs KO: 2ppm p=0.0684; 95% CI -1.669 to 11.46.
(C) 2ppm hematocrit: 8 week p=0.0028; 95%CI -12.04 to -3.005. 20 week p<0.0001; 95% CI -33.44 to -19.79
(D-E) Excel sheet
(F) %Splenomegaly p=0.0328; 95% CI -160.1 to -7.285
(G) Spleen population II+II: p=0.0129; 95% CI 0.6499 to 8.928
(H) Spleen RBC: p=0.0122; 95% CI 1.619 to 21.36
(I) BM RBC populations: V (p=0.1328; 95% CI -4.914 to 16.38), IV (p=0.3376; 95% CI -6.081 to 9.092), III (p=0.0457; 95% CI -11.75 to 0.9962), II (p=0.0495; 95% CI -15.19 to 1.481), I (p=0.0213; 95% CI 0.09237 to 4.633)
(J) RPMs: 2ppm WT vs KO: p=0.4511; 95% CI -1.015 to 0.9003
(K) Monocyte ratio: 2ppm WT vs KO: p=0.1787; 95% CI -0.1721 to 0.4589

eLife Sciences Publications, Ltd is a limited liability non-profit non-stock corporation incorporated in the State of Delaware, USA, with company number 5030732, and is registered in the UK with company number FC030576 and branch number BR015634 at the address 1st Floor, 24 Hills Road, Cambridge CB2 1JP | August 2014

Supplemental Figure 4

(A) In excel sheets

(B) BM RBC total: p=0.1616; 95% CI -6.686 to 19.2

Figure 5

(B) Excel sheet

(C) Spleen: Total p=0.0321; 95% CI -2872 to 79484, Organic p=0.0215; 95% CI 550.5 to 26667, Aqueous p=0.0794; 95% CI -1994 to 10238, Insoluble p=0.0397; 95% CI -4924 to 71436

(H) Non-Hemozoin: p=0.0279; 95% CI 0.1511 to 1.551, Hemozoin: p<0.0001; 95% CI 3.846 to 5.212

Supplemental Figure 5

(A) Liver: Total p=0.3766; 95% CI -38279 to 50855, Organic p=0.3560; 95% CI -11429 to 15976, Aqueous p=0.0614; 95% CI -26999 to 3894, Insoluble p=0.1122; 95% CI -10601 to 38834

(D) Non-Hemozoin: p=0.7505; 95% CI -0.1220 to 0.09535, Hemozoin: p=0.0388; 95% CI 0.003604 to 0.08240

(G) Lysosome diameter=0.0002; 95% CI 0.5999 to 1.797

Figure 6

(A) No t-test performed for the single data presented, but experiment was conducted at least twice resulting in the same observable difference.

(B) Basal p=0.1501; 95% CI -0.7359 to 3.355, 4h p=0.0009; 95% CI -16.80 to -8.807, 24h p=0.0681; 95% CI -9.897 to 0.5559

(C) 4h p=0.0475; 95% CI -17.37 to 3.079, 24h p=0.4903; 95% CI -7.016 to 4.729

(D) 4h Cells p=0.5358; 95% CI -26.61 to 43.76, 4h media p=0.5358; 95% CI -43.76 to 26.61, 24h Cells p=0.0353; 95% CI 2.368 to 39.98, 24h media p=0.0353; 95% CI -39.98 to -2.368

(F) For heterozygotes: (HRG1+/+ background Chi-sq= 0.09579, p= 0.9532), (HRG1-/- background Chi-sq= 14.63, p=0.0007)

(G) RPMs: p=0.0017; 95% CI -3.467 to -1.157

(H) BM RBC: p<0.0001; 95% CI -12.38 to -7.620

(I) BM RBC populations: V (p=0.0031; 95% CI -14.48 to -4.159), IV (p<0.0001; 95% CI -5.155 to -2.765), III (p=0.0038; 95% CI 1.928 to 7.072), II (p=0.0192; 95% CI 1.050 to 8.910), I (p=0.1568; 95% CI -0.2080 to 0.03997)

(J) Spleen RBC: p=0.0252; 95% CI -24.57 to -2.146

(K) Spleen population II+III: p=0.1947; 95% CI -3.932 to 16.43

Supplemental Figure 6

(D) RPMs: p<0.0001; 95% CI -2.479 to -1.975

(E) BMMs: p<0.0001; 95% CI -14.17 to 5.282

(F) BM RBC: p=0.8455; 95% CI -8.627 to 9.860

(G) BM RBC populations: V (p=0.4776; 95% CI -14.03 to 23.59), IV (p=0.4906; 95% CI -1.848 to 1.118), III (p=0.0482; 95% CI -6.028 to -0.04502), II (p=0.6422; 95% CI -15.32 to 11.05), I (p=0.0117; 95% CI 0.001346 to 0.005017)

(H) Spleen RBC p=0.2868; 95% CI -24.12 to 10.19

(I) Spleen population II+III p=0.0631; 95% CI -10.58 to 0.5111

(J) 4h Cells p=0.8587; 95% CI -40.84 to 35.61, 4h media p=0.8587; 95% CI -35.61 to 40.84, 24h Cells p=0.4888; 95% CI -6.180 to 10.85, 24h media p=0.4888; 95% CI -10.85 to 6.180

(For large datasets, or papers with a very large number of statistical tests, you may upload a single table file with tests, Ns, etc., with reference to sections in the manuscript.)

Group allocation

- Indicate how samples were allocated into experimental groups (in the case of clinical studies, please specify allocation to treatment method); if randomization was used, please also state if restricted randomization was applied
- Indicate if masking was used during group allocation, data collection and/or data analysis

Please outline where this information can be found within the submission (e.g., sections or figure legends), or explain why this information doesn't apply to your submission:

Samples were allocated into experimental groups based on the genotype of mice or cells. Masking was not used during group allocation.

Additional data files ("source data")

- We encourage you to upload relevant additional data files, such as numerical data that are represented as a graph in a figure, or as a summary table
- Where provided, these should be in the most useful format, and they can be uploaded as "Source data" files linked to a main figure or table
- Include model definition files including the full list of parameters used
- Include code used for data analysis (e.g., R, MatLab)
- Avoid stating that data files are "available upon request"

Please indicate the figures or tables for which source data files have been provided:

For HeatMaps generated based off quantitative PCR data, the software used is referenced in the experimental methods section.
